# Development and Validation of a Sensitive UHPLC-MS/MS Method for the Measurement of Gardneramine in Rat Plasma and Tissues and Its Application to Pharmacokinetics and Tissue Distribution Study

**DOI:** 10.3390/molecules24213953

**Published:** 2019-10-31

**Authors:** Nan Zhao, Hao-ran Tan, Qi-li Chen, Qi Sun, Lin Wang, Yang Song, Kamara Mohamed Olounfeh, Fan-hao Meng

**Affiliations:** School of Pharmacy, China Medical University, Shenyang 110122, China; Zhaonan-dd@163.com (N.Z.); thr9975@163.com (H.-r.T.); chenqili000@yeah.net (Q.-l.C.); jilindaxuesunqi@126.com (Q.S.); wanglin@cmu.edu.cn (L.W.); Songyanglhyb1998@163.com (Y.S.); Mohamedkamara6994@yahoo.com (K.M.O.)

**Keywords:** gardneramine, monoterpenoid indole alkaloid, LC-MS/MS, pharmacokinetics, tissue distribution

## Abstract

As a novel monoterpenoid indole alkaloid, gardneramine has been confirmed to possess excellent nervous depressive effects. However, there have been no reports about the measurement of gardneramine in vitro and in vivo. The motivation of this study was to establish and validate a specific, sensitive, and robust analytical method based on UHPLC-MS/MS for quantification of gardneramine in rat plasma and various tissues after intravenous administration. The analyte was extracted from plasma and tissue samples by protein precipitation with methanol using theophylline as an internal standard (I.S.). The analytes were separated on an Agilent ZORBAX Eclipse Plus C_18_ column using a gradient elution of acetonitrile and 0.1% formic acid in water at a flow rate of 0.3 mL/min. Gardneramine and I.S. were detected and quantified using positive electrospray ionization in multiple reaction monitoring (MRM) mode with transitions of *m*/*z* 413.1→217.9 for gardneramine and *m*/*z* 181.2→124.1 for I.S. Perfect linearity range was 1–2000 ng/mL with a correlation coefficient (r^2^) of ≥0.990. The lower limit of quantification (LLOQ) of 1.0 ng/mL was adequate for application to different preclinical studies. The method was successfully applied for determination of gardneramine in bio-samples.

## 1. Introduction

Gardneramine is a monoterpenoid indole alkaloid found in the plant species *Gardneria nutans* Sieb., *Gardneria multiflora* Makino, *Gardneria ovata* Wall, and other *Gardneria* species. [1,2,3,4,5]. *Gardneria* belongs to the Loganiaceae family and is widely distributed in east and southeast Asia, which is from India to central Japan and Java. All species can be found in China and were richest in Yunnan province [6,7]. Most of plants in this genus as traditional Chinese medicine, such as *Gardneria anguatifolia* Wall and *Gardneria multiflora* Makino, are employed to treat kidney deficiency, enuresis, aching lumbus and knees, wind-dampBi-syndrome, and injuries from falls [8].

Monoterpenoid indole alkaloids have long been a focus of natural product research because of their unusual carbon skeletons as well as potential bioactivities [9,10,11]. Gardneramine as an active monoterpenoid indole alkaloid has been shown to have excellent central depressive effect [12], to affect neuromuscular transmission [13], to produce a hypotensive effect like papaverine derived from peripheral vasodilatation, to have direct depressive action on myocardium, and to have central depressive action [14]. Previous studies indicated that gardneramine possesses a hexamethonium-like action on superior cervical ganglion and depresses the contraction of the organ through an inhibition of the parasympathetic ganglionic transmission [15,16]. Moreover, gardneramine was known to be an antagonist of nicotinic receptor by selectively inhibiting dimethylphenylpiperazinium-induced contraction [17]. These significant findings provide the basis for the further in-depth drug-targeted research of gardneramine as a novel anesthetic agent.

To our knowledge, the preclinical pharmacokinetic studies play a main role that can be used to make critical decisions supporting the safety and efficacy of the development of a new drug. In view of gardneramine as a potential anesthetic agent, it is of pivotal importance to understand its absorption and distribution in vivo. However, there is no report on the pharmacokinetics and tissue distribution studies of gardneramine in bio-samples. Therefore, in order to understand the characterization and diversity of gardneramine in vivo, it is also necessary and meaningful to establish a more accurate and selective bioanalytical method for the determination of gardneramine in plasma and various tissues.

Nowadays, UHPLC-MS/MS is a powerful technique used for drug research in vivo. Therefore, the aim of this investigation was to establish and validate a sensitive and credible approach for analyzing gardneramine in rat plasma and organs by using an UHPLC-MS/MS. Applying this method, we conducted the preclinical pharmacokinetics and tissue distribution characteristics of gardneramine in rats after intravenous administration.

## 2. Results and Discussion

### 2.1. Optimization of LC-MS/MS Conditions

**Figure 1:**
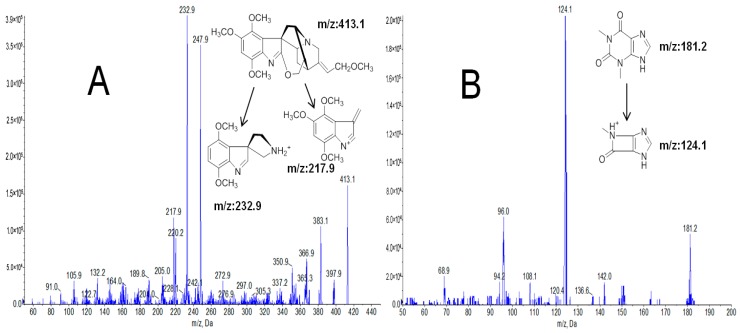
Mass spectra and structures of gardneramine (**A**) and internal standard (I.S.) (**B**).

To optimize MS/MS parameters, the feasibility of electrospray in both positive and negative ion modes were investigated. Gardneramine and I.S. were found to show higher response in positive ion mode. MRM mode was used to select the precursor ions and product ions, which were shown in Figure 1. A precursor ion and two MRM transitions have been established for gardneramine. The MRM transitions for gardneramine and I.S. were *m*/*z* 413.1→217.9 and *m*/*z* 181.2→124.1, respectively, which were employed for quantitative analysis. The qualifier ion for gardneramine was set at *m*/*z* 232.9. The parameters, such as declustering potential (DP) and collision energy (CE), were optimized to acquire higher sensitivity, which are shown in Table 1.

We also attempted to improve the mobile phase system in several trials. To obtain a satisfactory chromatograph, acetonitrile and 0.1% formic acid water were adopted as the mobile phase and column temperature and flow rate were optimized as 30 °C and 0.3 mL/min, respectively. The mobile phase additive, column temperature, flow rate, and run time were optimized. Finally, a gradient behavior was selected, and total run time was finished in 5.5 min.

### 2.2. Optimization of the Extraction Method

Protein precipitation and liquid–liquid extraction was considerably compared to select a suitable method for sample pretreatment. Ethyl acetate, dichloromethane, and tert-butyl ether as extracting agents produced low recovery and detectable interference from plasma matrix. Thus, protein precipitation was selected to perform the quantitative analysis. Methanol and acetonitrile were compared to screen a suitable solvent for protein precipitation, and deproteinization with methanol provided a high extraction recovery of more than 88.03% for gardneramine.

### 2.3. Method Validation

**Figure 2:**
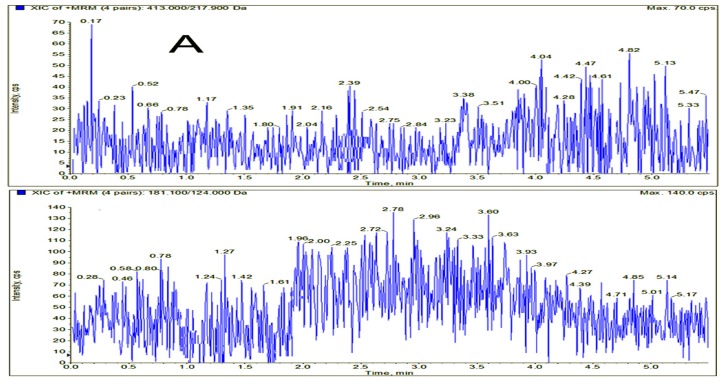

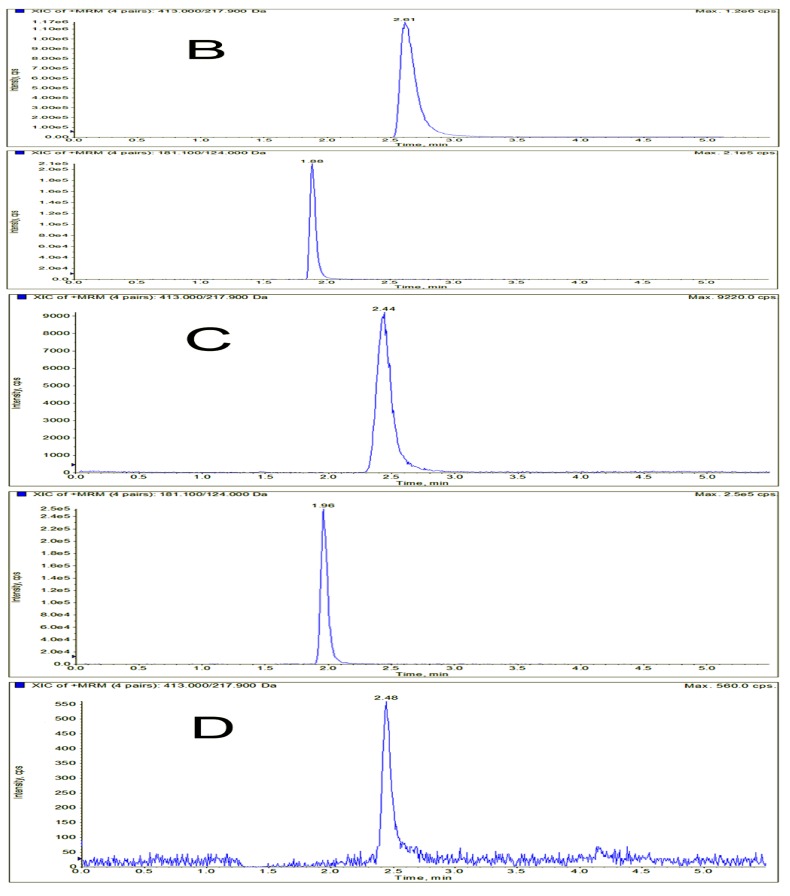
Typical chromatograms of gardneramine and theophylline (I.S.) in rat plasma samples: (**A**) blank rat plasma; (**B**) blank rat plasma spiked with gardneramine (2.61 min) and I.S. (1.88 min); (**C**) plasma sample from the pharmacokinetic study; and (**D**) blank rat plasma spiked with gardneramine at lower limit of quantification (LLOQ).

Typical MRM chromatograms of gardneramine and theophylline (I.S.) are shown in Figure 2, which were involved blank plasma, blank plasma spiked with gardneramine and I.S., and the plasma sample obtained from a rat 2 h after intravenous administration of gardneramine. Gardneramine and I.S. were eluted at retention times of 2.61 and 1.88 min, respectively. Under the validated HPLC-MS/MS conditions, no interference peaks were observed at the retention time of gardneramine and I.S. This result revealed that bio-samples could be accurately differentiated and quantified with this method.

The calibration curves, correlation coefficients, linear ranges, and lower limit of quantification (LLOQ) of gardneramine in the rat plasma and tissues homogenates were presented in Table 2. The calibration ranging from 1 to 2000 ng/mL yielded a good linearity (r^2^ > 0.990) corresponding to the range for gardneramine. The lower limit of quantification (LLOQ) for gardneramine was 1 ng/mL.

A summary of precision and accuracy of gardneramine in rat plasma and tissues is shown in Table 3. The data suggested that the precision and accuracy of the method were sufficient for the quantification of gardneramine in rat plasma and tissues.

The results of extraction recovery and matrix effects are presented in Table 4, which suggest that the endogenous matrix had no significant impact on the measurement of gardneramine in biological samples; the extraction recoveries of gardneramine at four levels were no less than 88%, indicating that the method of extraction we selected was dependable.

Stability data of gardneramine in rat plasma and different tissues are listed in Table 5 and show that gardneramine was stably detected in bio-samples stored under various conditions.

The results of dilution integrity experiments indicated that the accuracy of measured concentration was below 9.8 with a precision of no more than 3.8, which was acceptable in dilution integrity analysis.

### 2.4. Pharmacokinetic Study

**Figure 3:**
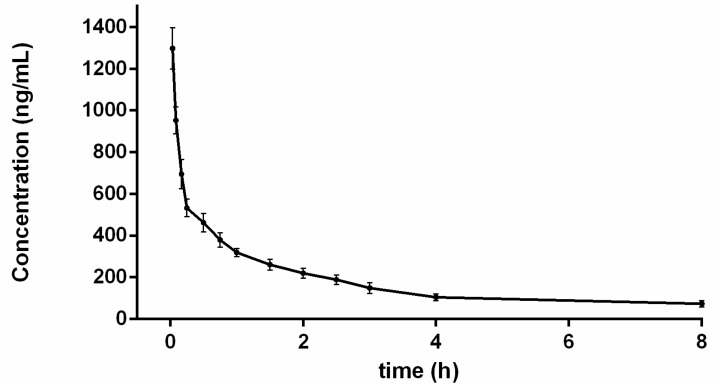
Plasma concentration-time profiles of gardneramine after intravenous injection of gardneramine at dose of 10 mg/kg (*n* = 12).

The validated UHPLC-MS/MS approach was successfully applied to pharmacokinetic study of gardneramine after intravenous administration of 10 mg/kg to rats. The mean plasma concentration- time profiles of gardneramine and major pharmacokinetic parameters are depicted in Figure 3. The pharmacokinetic parameters were estimated by using non-compartment model and are summarized in Table 6. After the intravenous administration of gardneramine in rats, elimination was fast with t_1/2_ of 1.94 h, indicating that the residence time of gardneramine was short. Moreover, rapid decline of the plasma concentration could be partly due to tissue distribution quickly, which was demonstrated by V_d_ of 19.30 ± 2.88 L/kg. V_d_ reflected the tissue uptake after intravenous administration, and the higher the value, the wider the distribution. At present, this is the first study about pharmacokinetics characteristics of gardneramine in vivo, and the observations suggested that the validated analytical UHPLC-MS/MS method was suitable and sufficient.

### 2.5. Tissue Distribution

**Figure 4:**
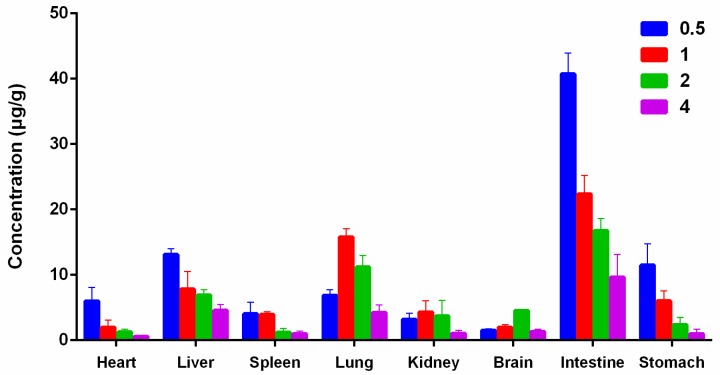
Tissue distribution in rats after a single intravenous administration of 10 mg/kg gardneramine (*n* = 5).

The established LC-MS/MS method was successfully applied for determination of gardneramine in rat tissues after an intravenous administration of 10 mg/kg gardneramine, as depicted in Figure 4. After intravenous injection, gardneramine underwent a wide distribution into all tested tissues, especially mainly into the intestine. The highest concentration of gardneramine was observed in the intestine (40.7 ± 3.2 μg/g, 0.5 h), followed consequently by intestine (22.3 ± 2.8 μg/g, 1 h), intestine (16.7 ± 1.8 μg/g, 2 h), lung (15.8 ±1.2 μg/g, 1 h), and liver (13.1 ± 0.9 μg/g, 0.5 h), which might have been attributed to the high blood flow in these organs. We speculate that these organs may be the targets of gardneramine and hypothesize a first rapid passage of gardneramine from the blood to the intestine and to the stomach partially, from which the substance would then be reabsorbed to reach the lung and kidney, where the concentration reaches a maximum after 1 h from administration. As we know, gut microbiota communicates with the central nervous system [18,19,20,21]. Gardneramine has a good ability to be absorbed in the intestine, suggesting that it perhaps influences the brain function and behavior by the gut-brain axis, which requires further researches. Meanwhile, the concentration of gardneramine in brain was increased within 2 h (4.5 ± 0.2 μg/g, 2 h) and then decreased in the following 2 h, which indicated that it could pass through the blood-brain barrier and did not accumulate during the detection period. The result was suggested that gardneramine could have potential to treat brain-related diseases, which was in coincidence with its central nervous activities. However, these findings require confirmation by further researches.

## 3. Materials and Methods

### 3.1. Chemicals and Reagents

Gardneramine (purity over 98%, by HPLC, Agilent Technologies, Palo Alto, CA, USA) was isolated from the roots of *Gardneria multiflora* Makino., and its structure was identified by detailed HR-MS and NMR analysis (Bruker, Karlsruhe, Germany). Theophylline as internal standard was purchased from Sigma-Aldrich (Shanghai, China). HPLC-grade acetonitrile and formic acid were purchased from Fisher Scientific (Tustin, CA, USA). Ultra-pure water, which was prepared by a Milli-Q purification system (Bedford, MA, USA), was used for the mobile phase.

### 3.2. Animals

Sprague Dawley (SD) rats (weighing 220 ± 20 g) were provided by the Experimental Animal Centre of China Medical University (Shenyang, China). All protocols and care of the rats were performed in compliance with the Guidelines for Care and Use of Laboratory Animals of China Medical University and authorized by the Animal Ethics Committee of the institution (code number: CMU2019202). The rats were acclimatized in an environmentally controlled breeding room in a 12 h light/dark cycle for one week at 22 ± 2 °C and 55 ± 10% relative humidity, with food and water provided ad libitum prior to testing, and then fasted with free access to water for 12 h before drug administration.

### 3.3. Instrumentation and Conditions

Samples were analyzed using an Agilent series 1290 UHPLC system (Agilent Technologies, Palo Alto, CA, USA) equipped with an AB 3500 triple-quadrupole mass spectrometer (AB Sciex, Ontario, Canada) and an electrospray source. The separation was carried out by using an Agilent ZORBAX Eclipse Plus C_18_ column (100 mm × 3.0 mm i.d., 1.8 μm) at 30 °C. A gradient elution program was performed using a mobile phase consists of acetonitrile (A) and 0.1% formic acid aqueous solution (B) as follows: 20–20% A (0–0.2 min), 20–95% A (0.2–2.3 min), 95–95% A (2.3–4.4min), and 20–20% A (4.5–5.5 min). The flow rate was 0.3 mL/min, and injection volume was 10 μL.

The mass spectrometer (AB Sciex, Concord, ON, Canada) was operated under multiple reaction monitoring (MRM) in a positive ionization mode using the following conditions: ion spray voltage of 5.5 kV, turbo spray temperature of 530 °C, gas 1 at 35.0 psi, gas 2 at 30.0 psi, curtain gas at 35.0 psi, and collision gas at 8.0 psi. The transitions (precursor-product) monitored were at *m*/*z* 413.1→217.9 and 413.1→232.9 for gardneramine and at *m*/*z* 181.2→124.1 for theophylline (I.S.). The optimized precursor and product ion-pairs and the mass spectrometer parameters are summarized in Table 1.

### 3.4. Preparation of Calibration Standards and Quality Control Samples

The stock solutions of gardneramine and I.S. were prepared by dissolving each reagent in methanol with the concentration of 1.00 mg/mL and stored at −25 °C. Calibration working solutions were achieved at 1.0, 5.0, 10.0, 40.0, 160.0, 400, 800, and 2000 ng/mL by diluting the stock solutions with methanol. The quality control samples were prepared at concentrations of 3.0, 45.0, and 1600 ng/mL for plasma and tissue samples. The intermediate stock solutions and working solutions were stored at 4 °C until use for no longer than 4 weeks.

### 3.5. Sample Preparation

The 100 μL of plasma sample, 50 μL of I.S., and 200 μL of methanol were added in 500 μL microcentrifuge tubes and then extracted by vortexing for 1 min to precipitate proteins. After a 10 min centrifugation step at 12,000× *g*, the upper organic layer was transferred to another tube and evaporated to dryness with a stream of nitrogen at 35 °C. The residues were reconstituted in 100 μL of initial mobile phase and centrifuged at 12,000× *g* for 10 min; 10 μL of the supernatant was transferred to sampling vials for the LC-MS/MS system.

The tissue samples (1.0 g) were homogenized in normal saline (5 mL). Homogenized tissues were centrifuged at 12,000× *g* for 10 min, and then, the upper layer was transferred to another tube. Subsequently, 100 μL of supernatant was treated in the same manner as the plasma sample.

### 3.6. Method Validation

Based on the FDA and EMA guidelines for bioanalytical method validation, the indicators of evaluation including selectivity, linearity, precision, accuracy, extraction recovery, matrix effect, and stability were followed for all experiments.

#### 3.6.1. Selectivity

The selectivity was assessed by comparing the chromatograms of blank rat plasma from six different sources, blank plasma-spiked gardneramine and theophylline, and actual samples after administration of gardneramine to investigate the potential interferences at the retention times of gardneramine and I.S.

#### 3.6.2. Linearity and Sensitivity

The calibration curves were generated by analyzing spiked calibration samples, ranging from 1 ng/mL to 2 μg/mL. The linearity of calibration curves was assessed by plotting the peak area ratio of gardneramine to I.S. versus the nominal concentration of gardneramine by weighted (1/x^2^) least square linear regression. The lower limit of quantification (LLOQ) was defined as the lowest concentration on the standard curve with a signal-to-noise (S/N) ratio > 5 and both the precision and accuracy <20%.

#### 3.6.3. Precision and Accuracy

The intra-day and inter-day precision and accuracy were evaluated by analyzing QC samples and LLOQ concentrations on three consecutive days (*n* = 6). Precision was calculated as relative standard deviation (RSD), while the accuracy was measured as relative error (RE).

#### 3.6.4. Extraction Recovery and Matrix Effects

For extraction recovery and matrix effect analysis, QC samples with three concentrations and LLOQ level were applied. Recovery was evaluated by comparing the responses of gardneramine from QC samples pre-spiked in blank sample with those of post-extracted blank plasma spiked at the same concentration (*n* = 5). The matrix effect was quantified by the comparison of the peak areas of post-extracted spiked rat plasma with those of equivalent concentrations of pure standard solutions (*n* = 5).

#### 3.6.5. Stability

Stability test of gardneramine was evaluated by examining three concentrations of QC samples with five replicates under different storage conditions: (1) freeze-thaw cycles stability (three cycles), (2) at room temperature for 4 h, (3) at 4 °C for 24 h, and (4) at −80 °C for 4 weeks.

#### 3.6.6. Dilution Integrity

The concentrations of gardneramine in some tissue samples are higher than the upper limit of quantitation (ULOQ). Experiments was performed by a 4.5-fold dilution of ULOQ concentration (*n* = 6), with both of accuracy and precision no more than 15%.

### 3.7. Pharmacokinetic Study

Gardneramine was dissolved in polyethylene glycol (PEG)-saline (1:4, PEG: 380–420) for the preparation of dosing solutions. Twelve SD rats (220 ± 20 g) were intravenously administrated with a single dose of 10 mg/kg. Blood samples (about 0.25 mL) were collected from suborbital vein of each rat at 2, 5, 10, 15, 30, 45, 60, 90, 120, 150, 180, 240, and 480 min after intravenous administration. The samples were placed in heparinized centrifuge tubes and immediately separated by centrifugation at 12,000× *g* for 10 min, and then, the supernatant fractions were transferred and stored at −20 °C until further analysis.

### 3.8. Tissue Distribution Study

Twenty rats were randomly divided into four groups (*n* = 5 per group), and intravenous dose was 10 mg/kg. Different tissues were harvested at each time point (30, 60, 120, and 240 min after administration) and prepared by the methods described in Section 3.5: Sample Preparation.

## 4. Conclusions

An accurate, sensitive, and efficient UHPLC-MS/MS method was established and validated for the measurement of gardneramine in bio-samples for the first time, and it was successfully used to evaluate pharmacokinetics and tissue distribution profile of gardneramine after intravenous administration to rats. The results suggested that gardneramine was well absorbed in plasma and all the other tissues tested in this study. Additionally, gardneramine can be easily taken up by intestine and brain, which revealed that gardneramine may be useful to the development of brain-related diseases therapeutics. Therefore, these findings will help to provide a reference for safe and effective drug doses for the further pharmacological and clinical practice of gardneramine and structural analogues.

## Figures and Tables

**Table 1:** Optimized mass spectrometry conditions for the determination of gardneramine and I.S.

Analytes	Precursor Ion (*m*/*z*)	Product Ion (*m*/*z*)	Declustering Potential (V)	Collision Energy (eV)
gardneramine	413.1	217.9	134.8	62.9
		232.9	123.9	46
I.S.	181.2	124.1	70	26.6

**Table 2:** Standard curves and LLOQ of gardneramine in biological samples.

Samples	Calibration Curves	Correlation Coefficients (r)	Linear Ranges (ng/mL)	LLOQs (ng/mL)
Plasma	Y = 0.0720 + 0.18447x	0.9924	1–2000	1
Heart	Y = −0.0943 + 0.13776x	0.9931	1–2000	1
Liver	Y = 0.0836 + 0.19987x	0.9906	1–2000	1
Spleen	Y = −0.0286 + 0.20747x	0.9913	1–2000	1
Lung	Y = 0.0996 + 0.09197x	0.9954	1–2000	1
Kidney	Y = 0.0193 + 0.25430x	0.9941	1–2000	1
Brain	Y = −0.0749 + 0.14529x	0.9917	1–2000	1
Intestine	Y = 0.0974 + 0.19646x	0.9962	1–2000	1
Stomach	Y = 0.0432 + 0.37274x	0.9901	1–2000	1

**Table 3:** Precision and accuracy of gardneramine in rat plasma and tissue homogenates (*n* = 6).

Samples	Spiked Concentration (ng/mL)	Intraday		Interday	
		Precision (RSD, %)	Accuracy (Mean%)	Precision (RSD, %)	Accuracy (Mean%)
Plasma	1	8.94	0.98	2.31	−1.38
	3	6.09	3.13	2.30	5.49
	45	4.89	1.33	6.27	4.29
	1600	5.80	3.65	3.47	4.89
Heart	1	3.05	1.02	4.03	4.21
	3	4.78	−1.42	3.75	−2.88
	45	4.53	1.33	4.08	3.18
	1600	3.88	3.79	2.81	3.88
Liver	1	3.64	1.02	3.27	3.62
	3	4.58	2.42	3.12	2.28
	45	2.57	0.33	2.13	0.17
	1600	3.96	0.53	5.07	−1.61
Spleen	1	3.34	0.33	7.10	−2.91
	3	3.07	−0.78	4.47	1.23
	45	2.24	−0.28	4.44	1.40
	1600	3.50	−1.39	1.04	−1.66
Lung	1	4.67	1.02	1.03	2.81
	3	3.56	2.84	2.24	−5.76
	45	1.69	−1.00	4.15	−2.26
	1600	3.50	1.84	3.14	2.87
Kidney	1	2.14	0.88	2.27	1.43
	3	2.74	3.55	4.96	5.53
	45	3.51	0.76	5.93	−1.72
	1600	9.59	2.32	4.38	4.77
Brain	1	4.47	1.03	2.64	3.96
	3	2.58	3.12	6.32	6.61
	45	4.86	−2.06	1.93	−2.77
	1600	10.24	0.47	4.07	−1.27
Intestine	1	4.76	0.92	7.77	−5.36
	3	3.26	−0.03	12.32	−4.68
	45	6.20	0.02	5.09	0.84
	1600	3.32	4.66	2.98	6.13
Stomach	1	4.55	0.16	3.50	−1.08
	3	5.27	3.20	2.40	8.17
	45	3.58	0.20	8.53	−2.49
	1600	11.08	−6.79	4.05	−8.04

**Table 4:** Matrix effect and extraction recovery of gardneramine in rat plasma and tissue homogenates (*n* = 5).

Samples	Spiked Concentration (ng/mL)	Matrix Effect		Extraction Recovery	
		Mean ± SD (%)	RSD (%)	Mean ± SD (%)	RSD (%)
Plasma	1	101.1 ± 5.8	5.8	101.6 ± 6.6	6.5
	3	101.4 ± 7.1	7.0	99.0 ± 7.5	7.5
	45	103.1 ± 6.9	6.7	93.5 ± 6.2	6.6
	1600	99.5 ± 4.1	4.1	99.5 ± 3.3	3.3
Heart	1	92.1 ± 3.5	3.8	94.0 ± 8.1	8.7
	3	99.8 ± 5.2	5.2	101.3 ± 5.9	5.8
	45	95.8 ± 6.1	6.4	99.9 ± 6.9	6.9
	1600	106.5 ± 9.6	9.0	95.3 ± 1.1	1.1
Liver	1	100.6 ± 1.3	1.3	99.5 ± 1.4	1.4
	3	102.0 ± 4.9	4.8	104.3 ± 3.7	3.5
	45	102.9 ± 1.9	1.9	99.6 ± 2.6	2.6
	1600	95.9 ± 3.4	3.5	102.6 ± 5.6	5.4
Spleen	1	96.3 ± 2.1	2.2	99.1 ± 2.3	2.4
	3	84.0 ± 3.5	4.2	111.1 ± 6.1	5.5
	45	113.05 ± 8.5	7.5	100.8 ± 9.9	9.9
	1600	111.4 ± 6.5	5.8	102.9 ± 4.4	4.2
Lung	1	99.9 ± 0.8	0.8	98.8 ± 5.0	5.1
	3	104.3 ± 2.7	2.6	103.1 ± 9.4	9.2
	45	101.2 ± 3.8	3.8	100.6 ± 3.9	3.8
	1600	100.8 ± 0.5	0.5	102.3 ± 3.7	3.6
Kidney	1	97.9 ± 3.6	3.6	101.5 ± 3.1	3.0
	3	95.6 ± 5.4	5.6	96.9 ± 5.4	5.6
	45	104.5 ± 3.9	3.7	99.4 ± 6.1	6.1
	1600	111.3 ± 2.0	1.8	91.6 ± 3.0	3.2
Brain	1	103.0 ± 9.1	8.8	95.0 ± 10.7	11.3
	3	106.2 ± 3.3	3.1	100.7 ± 5.9	5.9
	45	93.9 ± 3.5	3.8	103.3 ± 2.6	2.5
	1600	105.2 ± 1.0	1.0	100.4 ± 1.7	1.7
Intestine	1	100.8 ± 3.7	3.7	101.8 ± 5.5	5.4
	3	98.7 ± 1.5	1.5	88.0 ± 2.0	2.3
	45	100.3 ± 5.9	5.9	98.9 ± 4.0	4.0
	1600	96.0 ± 5.3	5.5	93.0 ± 4.5	4.8
Stomach	1	103.5 ± 3.4	3.3	100.0 ± 4.0	4.0
	3	94.6 ± 4.0	4.2	107.3 ± 1.6	1.5
	45	107.4 ± 5.9	5.5	105.9 ± 4.6	4.4
	1600	99.2 ± 5.2	5.3	105.3 ± 5.5	5.3

**Table 5:** Stability test of gardneramine in rat plasma and tissue homogenates (*n* = 5).

Samples	Spiked CONC (ng/mL)	Short-Term (at Room Temperature for 4 h)		Autosampler 4 °C for 24 h		Three Freeze-Thaw Cycles		Long-Term (at −20 °C for 30 days)	
		RE ^1^ (%)	RSD (%)	RE (%)	RSD (%)	RE (%)	RSD (%)	RE (%)	RSD (%)
	3	−10.2	6.62	−0.37	8.27	−3.45	5.06	12.8	4.55
Plasma	45	−4.78	1.50	−2.49	5.50	−1.72	5.64	1.37	4.01
	1600	−1.15	2.37	−1.56	3.69	−4.36	2.18	−1.11	7.07
	3	4.33	5.94	2.20	2.11	3.59	3.60	0.11	3.81
Heart	45	−11.7	2.60	−10.7	6.66	−7.75	4.44	4.49	3.37
	1600	0.77	3.32	−4.75	6.08	−4.89	2.25	−1.86	5.10
	3	−3.00	6.03	4.20	6.56	3.57	3.26	−2.75	6.10
Liver	45	1.76	3.19	0.66	3.75	5.36	6.22	−5.4	2.31
	1600	−2.10	2.99	2.09	2.32	−0.10	3.96	4.12	5.68
	3	−0.92	2.90	−4.67	5.49	−1.69	5.40	−7.62	2.38
Spleen	45	8.75	2.16	5.20	3.68	11.4	3.29	12.5	3.37
	1600	12.2	2.97	4.66	3.59	9.10	4.58	3.67	1.77
	3	7.71	5.70	7.86	3.79	9.81	3.23	3.03	1.13
Lung	45	0.94	7.00	2.15	5.19	6.06	1.60	−2.63	2.31
	1600	0.47	1.49	4.32	4.12	−1.53	2.97	0.59	5.68
	3	2.77	3.63	−8.30	2.61	−3.34	6.06	−8.27	2.52
Kidney	45	6.29	1.18	−4.53	8.27	−6.90	3.19	−8.88	1.96
	1600	−0.45	2.08	−10.36	4.59	6.73	4.85	0.49	3.49
	3	0.49	4.66	4.25	7.41	−4.07	2.50	−7.66	2.52
Brain	45	3.50	4.19	4.25	2.00	4.02	5.85	4.91	5.13
	1600	1.20	5.52	−1.89	6.80	3.52	1.85	9.09	4.41
	3	−4.88	4.45	−1.67	8.33	0.97	5.99	6.04	3.51
Intestine	45	−2.93	4.54	−6.52	4.54	−5.83	4.03	−9.83	5.02
	1600	−2.07	3.71	4.35	1.49	−2.42	5.78	−0.41	2.68
	3	−3.04	2.28	−3.46	1.76	−1.04	4.59	−0.18	6.49
Stomach	45	3.71	2.75	1.39	4.53	−2.04	4.88	6.23	2.24
	1600	−11.7	4.26	0.55	6.57	−11.3	6.76	−5.87	7.83

^1^ RE was relative error.

**Table 6:** The pharmacokinetic parameters of gardneramine in rat plasma (*n* = 12).

Parameters	Value
t_1/2_ (h)	1.94 ± 0.46
AUC_0–t_ (ng h/mL)	843.36 ± 54.02
AUC_0–∞_ (ng h/mL)	1443.88 ± 207.96
MRT_iv_ (h)	2.70 ± 0.64
CL (L/h/kg)	7.07 ± 1.00
V_d_ (L/kg)	19.30 ± 2.88
